# Electromagnetic-field theories of qualia: can they improve upon standard neuroscience?

**DOI:** 10.3389/fpsyg.2023.1015967

**Published:** 2023-06-01

**Authors:** Mostyn W. Jones, Tam Hunt

**Affiliations:** ^1^Retired, Washington, PA, United States; ^2^Department of Psychological and Brain Sciences, University of California, Santa Barbara, Santa Barbara, CA, United States

**Keywords:** qualia, EM-field theories of consciousness, images in cognition, cosmopsychism, general resonance theory

## Abstract

How do brains create all our different colors, pains, and other conscious qualities? These various qualia are the most essential aspects of consciousness. Yet standard neuroscience (primarily based on synaptic information processing) has not found the synaptic-firing codes, sometimes described as the “spike code,” to account for how these qualia arise and how they unite to form complex perceptions, emotions, *et cetera*. Nor is it clear how to get from these abstract codes to the qualia we experience. But electromagnetic field (versus synaptic) approaches to how qualia arise have been offered in recent years by Pockett, McFadden, Jones, Bond, Ward and Guevera, Keppler and Shani, Hunt and Schooler, *et cetera*. These EM-field approaches show promise in offering more viable accounts of qualia. Yet, until now, they have not been evaluated together. We review various EM field theories of qualia, highlight their strengths and weaknesses, and contrast these theories with standard neuroscience approaches.

## 1. The qualia problem and standard neuroscience solutions

### 1.1. The qualia problem

How is the external world in all of its many forms transformed into qualia in the mind? This is the “qualia problem.” This includes a “coding problem” because most standard neuroscience solutions ask how external phenomena are encoded in the brain through synaptic firing weights and related phenomena. Such encoding may not, however, be based solely on synaptic firing or “spikes.”

In this paper, we ask what are the physical mechanisms by which specific features of the objective external world are encoded into our subjective internal universe, our minds, with all of the informational and affective components that are included in each moment of consciousness? We discuss standard neuroscience approaches to this problem and then compare and contrast proposed solutions that rely on electromagnetic (EM) field theories rather than the more traditional neural and synaptic approaches.^1^

We flesh out different aspects of the qualia problem in §1.5.

Standard neuroscience is computational and focused on neurons and synaptic firing in terms of understanding how the brain and consciousness function. It faces the fundamental problem of explaining specifically how computations (information processing) and synaptic firings produce our consciousness–our privately experienced inner life of feelings, thoughts, etc., which is lost during comas and dreamless sleep. This consciousness is basically characterized by its qualia, which are our sensory and emotional qualities, such as pain and fear. It also has unity, which is exemplified by how various qualia are experienced as a whole when meeting an old friend. These unified qualia reflect the standard view (originally from Nagel, 1974) that consciousness is “what it is like” to, for example, smell a rose or to echolocate like a bat.^2^

Here in §1, we will analyze standard neuroscience’s primary problems in helping to explain how all the wide varieties of sensory and emotional qualia arise, and how they unite together. We will proceed from sensory qualia to unified sensory images, then emotional qualia.

In §2, we will investigate whether theories based on electromagnetic field rather than synaptic approaches can better explain the varieties of qualia we all experience than standard neuroscience theories. These electromagnetic approaches fit into various categories and are typically called “electromagnetic (EM) field theories of consciousness.” Numerous representative examples of these rapidly proliferating theories will be covered here, but we do not address those theories that neglect to address how unified qualia arise. We also address remaining issues with EM field theories and suggest some avenues for addressing these issues.

### 1.2. Problems with sensory qualia

Neuroscientists usually explain how our different sensory qualia arise in terms of specialized labeled lines with their own detector fibers and processing areas for taste, vision, and other sensory modes (e.g., Purves et al., 2001; Parker, 2019). Photoreceptors thus produce color qualia regardless of whether they are stimulated by light, pressure, or other stimuli. This method is supplemented by detailed comparisons of the fibers within each labeled line (e.g., Solomon and Lennie, 2007; Conway, 2009). For example, the three color fibers overlap in their response to short, medium, and long wavelengths of incoming light. So across-fiber comparisons of their firing rates help disambiguate which wavelengths are actually present.

This longstanding view has arisen from various historical roots. But the overall problem is that these operations are so similar in the visual, tactile, and other sensory modes that it is unclear how these methods can differ enough to account for all the stark differences between color and taste qualia, for example. Another issue (which will be addressed more below) concerns the “hard problem” of why this biological information processing is accompanied by any conscious experience of colors, pains, *et cetera*.

Such problems have not gone unnoticed by neuroscientists. For example, Humphries (2020) provides a book-length overview of the science of the “the spike code” (the synaptic firing approach that is the focus of standard neuroscience), particularly as it relates to visual perception. The book describes in detail how visual perceptions make their way from the retina through the central nervous system and coordinate with our motor control system. Humphries acknowledges, however, that neural spike activity and its relationship to consciousness remains largely unknown: “what we can predict are the new directions we want to explore. And what we want to explore is everything that is missing entirely from this book because we know nothing about them: spikes that underlie disorders of the brain, and spikes that underlie human thought processes.” He adds: “The most obvious chasm in our understanding is in all the things we did not meet on our journey from your eye to your hand. All the things of the mind I’ve not been able to tell you about, because we know so little of what spikes do to make them.”

It might be thought that recently proposed neuron-based neuroscientific theories of consciousness would offer more viable accounts of how different qualia arise. But they rarely do. For example, Global Neuronal Workspace Theory GNWT (e.g., Dehaene and Naccache, 2001; Dehaene, 2014) and Higher-Order Theories (e.g., Rosenthal, 2005) focus on access consciousness–the availability of information for acting, speaking, and reasoning. This access involves attention and thought. But these higher cognitive levels do not do justice to qualia, for qualia appear even at the very lowest levels of conscious cognition in pre-attentive iconic images (e.g., Koch, 2019). In contrast, Recurrent Processing theory (Lamme and Roelfsema, 2000) covers both access consciousness and phenomenal consciousness (the latter pertains to the subjective, qualitative feel characteristic of qualia). But this theory gives no account of how different qualia arise–so it is not covered in this paper.

Some prominent theories of consciousness that do address how different qualia arise include Integrated Information Theory–IIT (e.g., Tononi, 2008) and various quantum theories of consciousness. We start first with IIT.

IIT can be applied to any physical system and will provide a quantification of the capacity for consciousness in that system. But the authors of the theory look specifically to synaptic and related activity in the brain for an explanation of human consciousness (Hunt, 2020a, which is an interview with Christof Koch). IIT represents qualia information abstractly and geometrically in the form of a system’s “qualia space” (Tononi, 2008). This is the space where each axis represents a possible state of the system–a single combination of logic-gate interactions (typically involving synapses). Points along the axes are the probable efficacies of these various logic-gate combinations in the system. Arrows between the points represent information relationships between these elements. The overall set of information relationships constitutes the shape of the system’s qualia space, which in turn specifies the system’s experience. Thus, colors are different sub-shapes of the same kind (for example, pyramids pointing in different directions)–while sounds are very different sub-shapes (such as tetrahedra). Even the simple color of blue translates into a staggeringly complex shape in qualia space, for it must be differentiated not only from all other colors and all other perceptions, but also from all other experiences generally (*cf.* Aaronson, 2014).

IIT’s accounts of qualia spaces are far too complex to specify except in the simplest of cases, and no tests for this method of characterizing qualia has yet been proposed, as far as we are aware. This is unfortunate, for a useful theory of how different qualia arise needs to spell out the neural correlates of qualia in testable ways. But the difficulty in testing IIT–and its reliance on axioms, thought experiments, and abstract mathematical accounts–ultimately make this qualia theory seem in some ways less like an empirical hypothesis than a rationalist speculation. At least at this point in its development.

Other neuron-based theories that are (at least potentially) relevant to explaining qualia are the quantum-based theories of consciousness. To consider their potentials and shortcomings, let us start with the familiar example of the Orchestrated Objection Reduction theory (Orch OR), first suggested by Hameroff and Penrose (1996) and developed further in numerous papers. They argue that quantum states are coherent superpositions of microtubule states that incorporate many neurons when their electrons become entangled and inseparably correlated. (Microtubules are parts of neurons’ cytoskeletons which are important for maintaining cells’ shapes and intracellular transport, among other things) The collapse of these quantum states is attributed to gravity, and they are construed as being elementary moments of consciousness. One issue facing this approach to consciousness is whether quantum states can actually survive long enough in the brain’s thermal environment to affect cognitive mechanisms. Hameroff has addressed and dismissed these critiques in various papers (e.g., Hagan et al., 2002), but this debate continues.

Another issue here that is more relevant to this paper is that Hameroff and Penrose have not yet addressed how different qualia arise from different quantum states. This latter issue applies to many quantum theories of consciousness. They generally omit mention of how quantum states yield the primary sensory qualia (redness, sweetness, etc.) we are familiar with.

Some quantum-based theories do try to do this. But they remain problematic, in our view. For example, Beshkar (2020) contains an interesting QBIT theory of consciousness that attributes qualia to quantum information encoded in maximally entangled states. Yet this information ultimately gets its actual blueness, painfulness, etc. from higher cortical mechanisms criticized above and in §1.3.

Another example is Lewtas (2017). He also attributes our primary qualia to quantum levels. Each fundamental particle has some of these various qualia. Synchronized firing by neurons at different frequencies selects from the qualia and binds them to form images. This is ingenious, but binding mechanisms such as neuronal synchrony are problematic in explaining how pictorial images arise (see below). Far more detailed explanation is required here for how differences in neural mechanisms yield differ qualia.

Turausky (n.d.) posits a single quality in fundamental particles that contains all others. Just as visual qualia merge into whiteness, so all sensory qualia could merge into a neutral whiteout. Separate qualities differentiate out like a synthesizer filters an electric buzz to produce brass, string, or percussion melodies. These analogies are intriguing but hard to specify neurally.

The general problem with these highly philosophical qualia theories is that they are hard to evaluate. Their uniting of qualia to quanta is not spelt out in testable detail. Nor are quantum levels adequately tied to the existing neuroscience of perception. Typical quantum-binding theories have relied on synchronic and synaptic activity to explain why only certain neural assemblies support subjective experience (e.g., Da Rocha et al., 2001; Georgiev et al., 2007). If binding thus involves these synchronic and synaptic activities, why is quantum-level activity also needed to explain the overall coherence of neural activity and unity of sensory activity? In contrast, Eric Bond’s theory (below) is interesting in its attempt to address such shortcomings with an EM-field view of consciousness.

### 1.3. Problems with images

Having already looked at standard neuroscience’s problems in explaining how different colors (for example) arise, we will now turn to its problems in explaining how (1) colors get their shapes, and (2) how both bind together point by point to form a unified image. This is crucial to explaining qualia, for we actually perceive color qualia in the form of unified images, not as isolated points.^3^

(1) Our detection of the shapes in images starts with retinal activity and culminates with the ventral cortical pathway detecting ever larger borders, surfaces, and objects. It is often assumed that integrated shapes are perceived when higher neurons have sufficient information to detect which shapes are present together, as when three lines are seen as a triangle. While top-level cells do attentively monitor objects such as grandmothers (Le Chang and Tsao, 2017), no neurons monitor the entire visual scene (which is largely preattentive) to recognize the shapes present together in a particular scene. Indeed, it is impossible to have a top-level detector for each possible visual scene. So, while standard neuroscience has explained our perception of some shapes and objects, it has not yet explained our perception of overall shapes and layouts.

(2) Let us now turn to the binding of images into unified forms. Standard neuroscience has not explained well how the brain’s separate, distributed visual circuits bind together to support a unified image. This is an aspect of the so-called “binding problem” of how the mind’s unity arises (e.g., Treisman, 1998; LaRock, 2006, 2007).

This binding problem has several parts. To start with, visual processing uses separate, parallel circuits for color and shape, and it is unclear how these circuits combine to form complete images. Ascending color and shape circuits have few if any synapses for linking their neurons to create colored shapes. Nor do they converge on any central visual area (Zeki, 2003, p. 296; 1993, p. 216). Zeki may have overlooked here feedbacks from higher cortex into lower level maps (e.g., see Kawato, 1997; Lamme, 2004; Larkum, 2012). Arguably, these feedbacks might indirectly bind color and shape. But to encode detailed images, feedbacks would have to systematically connect shape and color elements point by point all across neural maps, which even the most detailed maps fail to do. [Nor is there any evidence of a central cortical area which higher cognitive functions connect into so as to account for the mind’s overall unity (*ibid*)].

Nor is binding wholly encoded by the firing of color and shape circuits in synchronized lockstep, as suggested by Dehaene and Naccache (2001), Dehaene (2014), Gray et al. (1989), Crick and Koch (1990), and Roelfsema et al. (1997). For example, Thiele and Stoner (2003), Dong et al. (2008), and others found that neural firing synchrony does not necessarily correlate with color and shape binding. Also, Koch et al. (2016) point out that some kinds of neural firing synchrony occur without consciousness, for example during anesthesia and seizures. Here hypersynchrony seems to disintegrate binding and normal consciousness disappears. So, there is inconsistent support for binding by neural-firing synchrony. (Below, we discuss how General Resonance Theory (GRT) explains binding via resonating/synchronized electromagnetic (EM) fields throughout the brain and body, instead of just synchronized neural firing in the brain).

Other lesser-known binding mechanisms are problematic too (Jones, 2017; Jones and LaRock, 2019). It is thus understandable that while IIT assumes conscious systems have unified causality (Tononi, 2008), it has not actually explained the mechanism that creates this unity.

So, standard neuroscience seems unable to explain the most basic level of cognition. It has not explained how the qualia, shapes, and unity of images are encoded. Nor does neuroscience adequately explain how such codes give rise to our conscious perceptions (as argued below).

### 1.4. Problems with emotional qualia

One of the best-known neurocomputational accounts of emotional qualia comes from Patricia Churchland (2014). She stresses their complexity. She says that the physiological functions of hormones are too numerous and complex to treat (for example) the function of oxytocin as simply being the love molecule. She instead attributes emotions to complex hormonal interactions. But she does little to actually specify these complex correlations.

Churchland (2014) approach partly resembles Lovheim’s (2012) hormonal approach to emotions. Unlike Churchland, he acknowledges (like many researchers) that serotonin correlates with the emotion of self-confidence, while dopamine correlates with anticipation and motivation, and noradrenaline correlates with distress. However, more in tune with Churchland, he treats varying levels of these three as the three axes of a computational space–a cube. These axes generate the cube’s eight corners, representing the emotions of anger, disgust, surprise, fear, joy, shame, excitement, and anguish–which Tomkins (1981) treated as the eight basic emotions. So, much like in Churchland’s view, the varying levels of the three hormones are assumed to be neural correlates of all these basic emotions.

However, this focus on basic emotions creates problems. Returning to the example of love, Lovheim offers no better guidance than Churchland on where it comes from. He presumably assimilates love with Tomkins’ emotion of joy. But the joy of romantic love differs greatly from the joy of monetary riches or the joy of children playing. Arguably, his preoccupation with the “basic” emotions such as excitement, joy, and surprise misleads him into thinking that joy is a single emotion with a single cause. He ignores the rich variety of both emotions and hormones.

Moreover, there is little evidence that all these various emotions correlate in any systematic way with varying mixtures of the three hormones that Lovheim’s computational emotional space so narrowly focuses on. It is thus most likely that mixtures of hormones instead just affect (for example) love quite indirectly by modulating levels of oxytocin in limbic circuits and thus intensities of love feelings. (See §2.5 below for a noncomputational view of qualia like this one).

These issues demonstrate, at the least, that these issues with emotional qualia are not explained or tested in any significant detail in the standard neuroscience paradigm.

### 1.5. Standard neuroscience’s three main qualia problems

The main problems above in neuroscience’s accounts of qualia seem to fit into three categories. These problems actually apply to all accounts of qualia–neuron-based, EM-based, computation-based, *et cetera*. But they will be cast here initially in terms of the standard neuron-based and computation-based accounts above. This focus will help to summarize the problems in neuroscience and (in some cases) further sharpen critiques of it.

(1) *The coding/correlation problem:* As argued above, the neuronal and computational accounts above have failed to find different information-processing operations among neurons that encode our different qualia. More generally, this issue concerns how to specify the various neural correlates of qualia, whether or not they are computationalist. But sticking now to neuroscience’s current computational approach, this issue may arise simply because these encoded operations are highly elusive. Alternatively, it may arise because qualia are ultimately not computational and neuronal in character. The next two qualia problems together suggest that the latter may be true.

(2) *The qualia-integration problem*: Computational accounts also face the problem of explaining how myriad qualia are integrated together to produce overall unified perceptions such as visual images. Detector neurons are buried in visual circuits and have only limited localized information. So, each detector neuron lacks the global perspective needed to create an overall, unified picture (Van der Velde and de Kamps, 2006). This integration could instead come from systematically connecting cells hierarchically via synapses, gap junctions, *et cetera*. For example, as already noted, some ventral-cortical detectors connect into many lower detectors to recognize particular objects, such as faces. Yet there are no top-level detectors to recognize all possible visual scenes. So, this circuitry has isolated information about different shapes but no unified, global perspective. Similarly, color and shape circuits do not synapse systematically, so their cells also lack global perspectives for integrating the circuits. Here the integration problem dovetails with the binding problem concerning what mechanism is uniting colors and shapes into an overall pictorial image. Neuroscience has yet to find any specific neural codes for this unified pictorial form.

(3) *The hard problem:* In addition to the two empirical problems above, computational accounts face a hard, metaphysical problem. Why are neural events accompanied by any qualia at all? That is, are the two related by identity, causality, third entities–or some other relation?

To start with, computationalist (information-processing) accounts of cognition treat minds as abstract computing systems that are realizable in multiple hardwares or substrates (Rescorla, 2015). Examples are Putnam’s (1967) computational functionalism, Churchland’s (1986) computational qualia spaces, and Tononi (2008) IIT (see Koch, 2019, p. 150).

The problem here is that images are qualities that we experience. In contrast, computations are mere abstract relations. They are abstract for two reasons. (a) Since computations are said to be multiply realizable in different hardwares, they are abstracted from any particular hardware. (b) Haugeland (1985) argued that the blind, mechanical activities of hardwares only become meaningful information once we impose high-level, abstract functions on them (facial recognition, language translation, etc.). So, information states are necessarily abstract, theoretical constructs in the minds of scientists.

So, computationalist claims that images are just neural computations face an important explanatory gap. There is no such gap when we explain (for example) how temperature is just kinetic energy. But images are so radically different from the abstract relations comprising computations that the latter fails to explain the former (e.g., Levine, 1993). So, while questions about what computations correlate with images involve “easy” empirical problems, questions about how images and computations are related involve a “hard” metaphysical problem (Chalmers, 1996). The radical differences between computations and images not only make it hard to treat them as identical, but also to posit any possible causal relation between them, so it is hard to see why qualia accompany neural computations. For example, the emgergence of conscious images from organized brain activity that lacks consciousness seems like sheer magic. Relations of aspects, realization, grounding, etc. are obscure for much the same reason (Jones, 2016). Computationalists end up with three quite different entities–images, neurons, and computations–with obscure relations between all three.

Computationalist theories will appear again below. Ultimately, in §2.4–2.5, we will reconstrue “information” and “computations” in terms of concrete and measurable EM activity between neurons (versus abstractions). We will also try to attribute qualia to this concrete EM activity without any overt explanatory gaps, so as to avoid the problems just listed here.

## 2. Electromagnetic field theories of qualia

**Figure 1:**
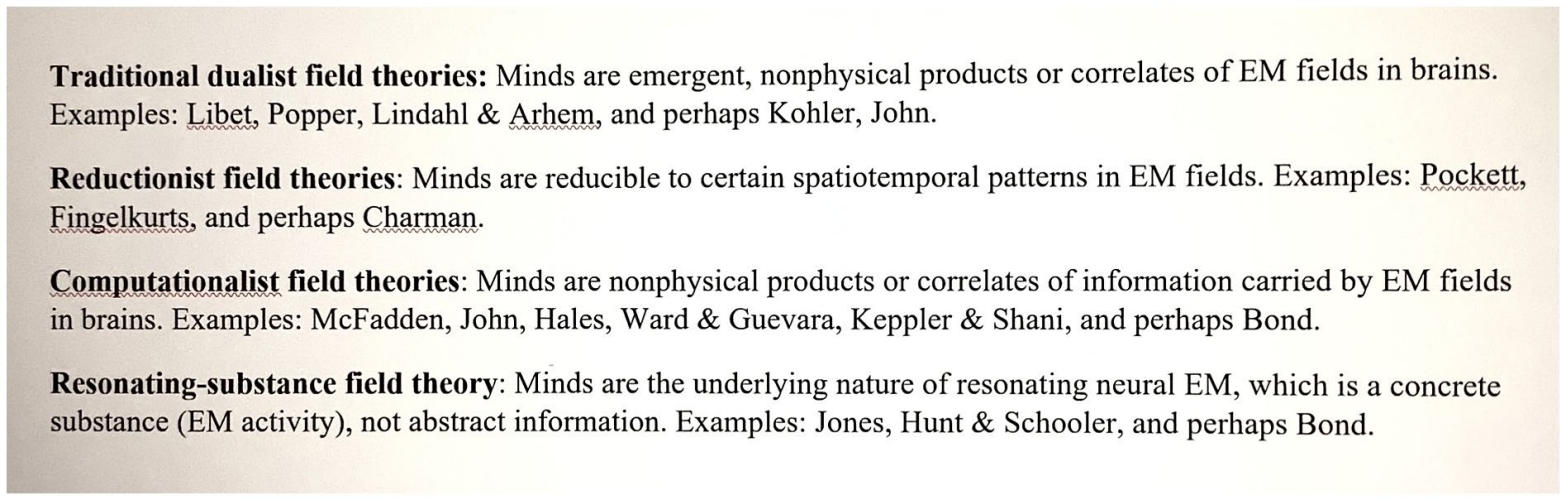
Varieties of EM-field theories of consciousness the traditional dualist theories do not address how different qualia arise. So, they do not appear in the present paper.

While standard neuroscience seems stymied in explaining how brains create our different qualia and unify them into phenomenal consciousness, EM field approaches to minds have offered new theories of qualia and consciousness, some of which are testable. These electromagnetic approaches seat consciousness primarily in the various complex EM fields generated by neurons, glia and the rest of the brain and body. They can be classified in the varieties listed at the end of this paper in Figure 1 (see Jones, 2013, for references and reviews). The classifications overlap at times. They differ in their definitions and (for example) whether the qualia they attribute primarily to EM field activity are global or localized in brains, whether field-brain causality is one-way or two-way, and whether qualia are more akin to substances or to information.

These EM field approaches are proliferating because they draw on considerable experimental evidence and withstand past criticisms from standard neuroscience. For example, they have explained the unity of consciousness in terms of the physical unity (by definition) of EM fields–in contrast to the discrete nature of neurons and their synaptic firing. In the last two decades, they have also offered explanations of how neural EM activity creates different qualia.

### 2.1. Qualia as global EM activity in brains

Pockett’s (2000) theory of qualia is an important landmark in EM field theories of mind. It is rooted in extensive experimental evidence, makes testable predictions, and is strongly defended against critics. If Kohler, Libet, Eccles, and Popper helped establish the EM field approach to minds, Susan Pockett has arguably done more to develop it than anyone else–except for perhaps Johnjoe McFadden. In this section, we will start with Pockett, then end with McFadden. Both attribute qualia to EM field patterns, though they differ in their metaphysical views on how exactly these patterns are related to qualia. Pockett arguably tends more toward treating qualia as concrete patterns in field substances, while McFadden treats them as abstract field information.

#### 2.1.1. Pockett’s theory

Pockett’s basic claim is that “consciousness is identical with certain spatiotemporal patterns in the electromagnetic field” (*ibid.*, pp. vi, 109, 136–7). Her evidence comes mainly from extensive EEG and MEG studies of neural electromagnetic fields. They show correlations between sensory qualia and field patterns. For example, EEG studies by Freeman (1991) show that various odors (e.g., from bananas or sawdust) correlate with specific spatial patterns distributed across mammalian olfactory areas. The patterns altered when animals were trained to associate the odors with rewards, showing that the correlations were with odor awareness, not just chemical stimuli. Given the problems with neuronal correlates with qualia above, these global EM patterns appeared as more promising physical correlates of qualia.

Similar correlations appear in Freeman’s many studies of auditory and visual awareness. Also, EEG studies by Laurent et al. (1996) show that these sorts of spatial patterns evolve while odors are puffed onto locust antennae. So, Pockett thinks that fields create a specific spatiotemporal pattern for each kind of sensory quality. This can ultimately be tested by examining whether these trends persist through all sensory and emotional qualia. Pockett (2011) attributes these qualia even to possible electromagnetic fields created artificially outside brains.

Pockett’s (2012) theory not only offers a testable EM theory of how different qualia arise, it also offers a way to distinguish nonconscious fields from conscious fields. Assuming that the latter reside in the cerebral cortex (which has a six-layered architecture), she suggests that “conscious fields will have a surface layer of negative charge above two deeper layers of positive charge, separated by a distinct neutral layer”. The fields are boosted to these significant levels of electrical activity by synchronized feedback between cortical areas (*ibid*). Also, modes of consciousness and individual experiences will reside (respectively) in regional variations in cortical thicknesses and cortical modules (*ibid.*). She gives various kinds of evidence for all these points while acknowledging that they just provide necessary conditions for consciousness (*ibid.*, §5). Pockett’s (2012) analyzes of these conscious patterns suggests that electromagnetic fields may be the only conscious fields.

Pockett stresses that experiences are distributed across the brain’s global electromagnetic field. For example, our perception of a red spot is widely spread across this field—it is not in one place (e.g., Pockett, 2000, pp. 10–11, 65–7, 70, 108). The field binds the spot’s color, shape, and motion into an overall experience (*ibid.*, pp. 107–8). Images reside in global fields in nonpictorial, coded forms.

Pockett’s theory raises issues that appear in other theories of qualia as well as her own. To start with, she realizes the problem raised by traditional identifications of qualia with firing neurons–given their observable differences. Yet, intriguingly, she feels that this problem is lessened by instead identifying qualia with the brain’s “everchanging, shimmering, invisible” electromagnetic field (*ibid.*, pp. 136–7). She seems closest here to the reductionism of psychoneural identity theory (Pockett, 2000, pp. 109, 135–6; but *cf.* pp. 105, 136).

Yet since qualia cannot be observed by investigating this everchanging electromagnetic field, their identity remains problematic. Here, Pockett faces the same explanatory gap between the mental and neural that bedevils all attempts to fully explain minds in terms of physics. But Pockett’s thoughtful psychoneural identity theory might be a step in the right direction here. For qualia might be private because they are hidden from public view (“invisible”) in the sense that they are the underlying nature of fields that we detect only *indirectly* via EEGs. Explaining in this way why color qualia are not observable in neural activity could arguably help deal with the explanatory gap between color qualia and neural activity, for both the colors and the EM fields are not directly observable in brains (see §2.5 below).

It should be noted that while Pockett (2000), pp. vi, 109, 136–7 espouses a reductionist “psychoneural identity theory” in which “consciousness is identical with certain spatiotemporal patterns in the electromagnetic field,” this needs qualification. She also repeatedly mentions neural information processing, though its relation to consciousness is not made clear. So it is possible that she is reducing qualia to either the physical substance of EM field patterns or the information they carry. Nonetheless, information approaches are typically nonreductive. So, we will construe her identity theory as attributing qualia to EM field’s physical substance instead of their information.

Another possible objection concerns Pockett’s view that the experiences of qualia are widely distributed across the brain. While Freeman found that each olfactory stimulus creates widely distributed responses in the olfactory system, other studies show that such stimuli create strong, isolated (versus global) responses (e.g., Stewart et al., 1979; Jones, 2010). Actually, the strongest responses in Freeman’s own studies are rather isolated too–arguably his weaker responses are largely from the proclivity of detectors to respond faintly to diverse stimuli.

There is evidence that only this strong kind of sensory activity is fully conscious, while the rest is weakly conscious or subliminal. For example, it is widely known that qualia intensity covaries with the number and rapidity of neurons firing in sensory pathways. Also, MEG studies show that electrical activity is far higher in fully conscious processing than in the subliminal processing of binocular rivalry (Edelman and Tononi, 2000).

Arguably, such evidence might support treating perceptions as localized events where an image–for example, a yellow spot–is not widely distributed. Instead, it appears when one type of wavelength detector is most strongly active at a spot in retinas and associated sensory maps. This fixes the spot’s color, shape, and location in the image.

By contrast, Pockett’s global field theory is unclear on how globally distributed yellow spots would get their actual locations in images. If the field’s spatial patterns are used to specify which colors exist, then what is left to specify the colors’ spatial locations in images? This is part of a larger problem in computational approaches to minds, namely, how can nonpictorial field patterns be identified with pictorial images?

So, Pockett has important ideas about how qualia are created, which fields are conscious, *et cetera*. Her defense of field theory is also sophisticated. Yet questions may arise about how experience is identical to fields and how fields unite colors and shapes into all their right locations in pictorial images. To be fair, these questions apply not just to Pockett, but to other theories too.

#### 2.1.2. McFadden’s theory

As promised, we will now turn to McFadden’s theory with an eye toward comparing it to Pockett’s theory. McFadden’s theory is the leading EM-field theory of consciousness today. It says that information is conscious at all levels, which seems to entail a form of panpsychism (McFadden, 2002b). The “discrete” consciousness of elementary particles is limited and isolated. But as particles join into a field, they form a unified “field” consciousness. As these fields affect motor neurons, the brain’s consciousness is no longer an epiphenomenon, for its volition can communicate with the world. This level of “access” consciousness serves as a global workspace where specialized processors compete for access to volition’s global, conscious processes (McFadden, 2002a, 2006).

McFadden (2002a, 2006) cites evidence that fields affect nerves, as the last level stipulates. For example, transcranial magnetic stimulation (TMS) produces fields as strong as the brain’s own native fields, and these TMS fields make nerves fire. Field–nerve interactions occur mainly when fields are strong due to synchronized firing in regularly aligned nerves, and when nerves are myelinated and bent relative to field isopotentials (McFadden, 2002b). This affects neurons poised near firing thresholds, which proliferate when we are undecided (McFadden, 2006).

As noted above, McFadden rejects popular views that minds are just ineffectual epiphenomena of brain activity. Instead, field–nerve interactions are the basis of free will. The conscious field is deterministic, yet it is free in that it affects behavior instead of being epiphenomenal (McFadden, 2002a,b). This treats determinism as compatible with free will construed as self-determination.

McFadden (2002b) concludes that “Digital information within neurons is pooled and integrated to form an electromagnetic information field. Consciousness is that component of the brain’s electromagnetic information field that is downloaded to motor neurons and is thereby capable of communicating its state to the outside world.” He calls this theory “The conscious electromagnetic information” (CEMI) field theory.

McFadden has not said as much about qualia as Pockett, for he feels that detailed accounts of qualia are not possible, given our current knowledge. Yet his 2002a paper has an entire section on qualia. Like Pockett’s theory, this paper attributes different qualia to different field patterns, such as those discovered by Freeman (1991). But McFadden stresses that these qualia arise only when processing streams are well integrated in brains due to the neural EM field. Reiterating this overall view, McFadden (2020) says, “the qualia associated with hearing the musical note middle C is what an EM field perturbation in the brain that correlates with the sensory input of middle C feels like, from the inside.” Yet unlike Pockett (2000), he does not feel that these patterns are close to being specified at this time.

Like Pockett, McFadden (2002a) addresses the hard problem of why such field patterns are accompanied by qualia. But he does not adopt her psychoneural identity theory, where qualia are outright identified with neural field patterns. Instead, he adopts a functionalist approach that ties qualia to the functional organization of neural activity. Here he mentions well-known arguments that if qualia come from functional or computational organizations, then the population of China could at certain times have qualia—or qualia would fade away if brain circuits were gradually replaced by silicon chips. Turning to his own CEMI, McFadden says here that if brain circuits were gradually replaced by silicon chips, this would not produce qualia unless the neural EM field was somehow preserved in this replacement process (Pockett expressed a similar view above).

So, McFadden ends up with a functionalist-computationalist metaphysics quite different from Pockett’s psychoneural identity theory. McFadden says that phenomenology (the study of consciousness) describes information from the inside, where it is privately experienced, while physics describes information from the outside, where it is physically observed (McFadden, 2002a,b). This echoes Chalmers’ neutral monism, where the basic stuff of the world is not mental or physical, but neutral. The mental is constructed from its inner, intrinsic nature. The physical is constructed from its outer, extrinsic relations (Chalmers, 1996, pp. 155, 305).

To summarize, McFadden’s theory of qualia resembles Pockett’s theory that qualia correspond to neural EM-field patterns, though he is less sure about specifying these patterns. Also, McFadden explains why these correspondences obtain in terms of a different metaphysics than Pockett’s. He relies on abstract functional–computational links between qualia and field patterns, while she seems to rely more on an outright identity between qualia and field patterns–that is, between qualia and physical field patterns versus abstract field information. Her view is a monism in which qualia just are neural field patterns, while his is more of a dual-aspect view in which qualia and field patterns are different aspects of information. Both theories are sophisticated empirically and metaphysically. Yet, like all extant theories of qualia, they raise certain issues.

#### 2.1.3. Field theory’s three main qualia problems

The three kinds of qualia problems we found in standard (neuron-based, computation-based) neuroscience also apply to the first two EM-field theories of qualia we have just reviewed above.

(1) *The coding/correlation problem*: What different EM-field activities encode or correlate with the various qualia? Both field theories above face difficulties here. Pockett’s psychoneural-identity theory is based on correlating qualia such as colors with the spatial patterns of fields. But, as already noted, this makes it hard to specify the colors’ spatial locations in images. McFadden’s computationalist field theory above (and other computationalist theories below) are less forthcoming than Pockett’s theory when it comes to spelling out correlations between qualia and information-processing operations that would encode these qualia. Arguably, these field theories have a way to go here before they can be said to improve upon standard neuroscience.

(2) *The qualia-integration problem:* How do EM fields integrate myriad qualia to form (for example) unified pictorial images? Here field theories seem quite promising in their ability to improve upon standard neuroscience.

As already noted, one aspect of this integration problem is the binding problem. The latter arises because standard neuron-based neuroscience has not shown how qualia bind together to form unified images by synaptic connections or synchronized firing of neurons. In contrast, field-based theories can attribute binding to the field’s substance, which is a continuous, unified conscious whole. This field can thus pool different qualia together in the same consciousness. This is, quite arguably, an important advance upon standard neuroscience.

Nonetheless, computationalist field theories still face another aspect of the integration problem. In EM fields, how does information about qualia integrate together to encode or construct unified pictorial images? Fields pervade visual circuitries, so it might seem that they can readily connect information about particular colors together to form a more complete colored image. But this pervasiveness arguably leaves fields unable to selectively connect colors together in systematic ways. In contrast, neurons could in theory use synapses to selectively connect color information together point by point across neural maps (though they evidently do not do so, as already noted). But an EM field arguably lacks these selective connections and instead just pools color information together as a whole into a single consciousness.

Pockett’s theory is an example. Once again, it uses the overall spatial form of fields to specify the colors in images. So, what is left to specify the colors’ spatial locations in images? What we are left with are field patterns that do not appear anything like our pictorial images—and do not seem to explain how myriad colors combine point by point to form pictorial images.

It may seem that there is no real problem in explaining how colors are integrated to form pictorial images Arguably, neuroscientists are already showing how brains encode and construct such images. They are using artificial intelligence to decode patterns in EEGs and partly reveal people’s visual images of (e.g.) faces. Or so it may seem. In fact, these EM patterns just arise from the processing that recognizes faces in terms of their gender, familiarity, etc.–which is quite different from the processing that actually constitutes the image in the first place. (Even if these coded patterns helped constitute images instead of just recognizing images, the question arises of how brain mechanisms would go about decoding these complex codes to yield our actual images—this issue leads into the hard problem below.)

So, field theory still owes us an explanation of how integrated pictorial images arise. It has not yet shown how fields integrate colors together point by point to create images. Arguably, one remedy is to attribute the colored spots in images to highly localized fields in neural maps that are rooted in retinas. Here, specific colors would come from color detectors’ EM activity in maps and the colors spatial position in images would come from the detectors’ spatial positions in the maps.

This would offer a simple way of connecting colors together in a pictorial form (see §2.5 below). But it is unclear whether computationalists would accept this, for the pictorial form of images is no longer a coded space–it is the actual space of neural maps. Computationalists may choose instead to wait patiently for the discovery of purely coded spaces and images akin to those in the EEG studies just mentioned above.

So, field theories have quite arguably improved on standard neuroscience in explaining how qualia bind together into unified forms. But it is currently unclear which direction field theories will take in explaining how colors integrate to form pictorial images–and whether they can improve on standard theories here.

(3) *The hard problem*: Apart from the issues above concerning neural correlates of qualia and their integrations into images, field theories face a relatively hard metaphysical issue. Are fields metaphysically related to qualia by identity, causality, third entities–or some other relation? The two theories above arguably construe qualia in terms of patterns in concrete field substances–or in terms of abstract field information. So, they arguably face similar problems to those in many other theories concerning how qualia can be intelligibly related to concrete neural substances or abstract computations or by relations of identity, causality, aspects, realization, grounding, third entities, etc. (Jones, 2016). For example, information approaches end up with three radically different entities (information, qualia, and EM) with obscure relations between each. In the end, metaphysical theories associated with field theories and standard neuroscience seem to be in the same boat. So, field theories have not improved on the latter in this regard.

### 2.2. Qualia as localized EM field activity in brains

In contrast to Pockett’s global qualia in specific kinds of EM fields, Ward and Guevara (2022) localize qualia in the fields generated by a particular part of the brain. Their intriguing thesis is that our consciousness and its qualia are based primarily on structures in thalamic EM fields which serve to model environmental and bodily information in ways relevant to controlling action.

Ward and Guevara argue that the physical substrate of consciousness is limited to strong neural EM fields where synchronously firing neurons reinforce each other’s information (instead of randomly firing neurons canceling each other out). They qualify this by adding that epileptic seizures are nonconscious even though they involve strong, synchronous firing. They thus contend that more is required for consciousness, namely, that fields also be integrated and complex.

Ward and Guevara adapt other views from field theories of consciousness. For example, they say that these EM fields contain all the information carried by the fields’ neuronal sources. Also, these fields are integrated at light speed while neurons’ synaptic integrations are relatively slow. Finally, local, nonsynchronous fields can be canceled out in favor of a dominant field that synchronously and coherently represents all the information from our senses, memories, emotions, *et cetera*. For these reasons, Ward and Guevara believe that fields are better candidates than neurons and synaptic firing for the primary substrate of consciousness.

Much like John (2001), they attribute consciousness to a specific part of the brain’s EM field. They stress that this contrasts with attributing consciousness to the brain’s entire field (as in Pockett, 2000 and Hales, 2014, for example) or to a specific kind of brain activity involving fields (as in McFadden, 2002a,b; McFadden, 2020) or to a nested hierarchy of EM fields in the brain (as in Fingelkurts et al., 2010, 2013; Hunt and Schooler, 2019). In defending this view, Ward and Guevara argue that (in mammals) the field’s conscious part is generated by the thalamus.

Following Ward (2011), they cite four reasons for ascribing consciousness to the thalamus. (1) We are not conscious of all sensory computations, just their end result, which involves the thalamic dynamic core. (2) Thalamic dysfunctions (but not necessarily cortical dysfunctions) are deeply involved in nonconsciousness conditions such as anesthesia, unresponsive wakefulness syndrome, and anoxia. (3) The thalamus is a prime source and controller of synchronization (in itself and in cortex), which is also associated with consciousness. (4) The thalamus (especially its DM nucleus) is ideally suited for the integrative role associated with consciousness, for cortical feedbacks seem to download cortical computations into thalamus. All this aligns with suggestions that thalamus serves as an attentional searchlight during perception (Crick, 1984) and as an active blackboard for offering best guesses while representing objects (Mumford, 1991)–but see below. These lines of evidence indicate that while cortex computes qualia, thalamus displays qualia. Ward and Guevara contrast this view with Fingelkurts et al. (2010, 2013), where the highest of various nested levels of consciousness contains the conscious features of the lower levels. Ward and Guevara say that this does not explain why only the end result of information processing (which results from cortical feedbacks into thalamus) is conscious.

Ward and Guevara then argue that sensory qualia are the EM field structures of the thalamic nuclei, which model the information structures of the environment and sensory systems. For example, the thalamic field contains information from the retina and higher-level retinotopic maps about color, motion, shape, and the overall topology of the visual field. Their idea here seems to be that visual colors differ from auditory pitches because their information structures differ in the thalamic EM field (this idea thus resembles McFadden’s computationalism more than Pockett’s identity theory above). This partly resembles IIT’s large-dimensional qualia space in which qualia are represented by vectors. But Ward and Guevara do not spell out these qualia spaces as IIT does.

Ward and Guevara identify consciousness in general with this EM field of the thalamic dynamic core which reflects the environment in ways relevant to controlling action. They reiterate what it is about this field that is conscious. It is not just the field’s strength and synchrony–for smaller animals such as birds lack this strength yet still seem conscious. Also important is the complexity and differentiation-integration of the field’s information, as already noted. Finally, the field is “unitary and reinforcing” relative to isolated fields in other areas that cancel each other out.

Ward and Guevara ask why we should equate qualia with a neural EM field rather than with, for example, Edelman and Tononi’s (2000) differentiated and integrated neuronal activity. Their answer builds on their argument above that fields are better candidates than neurons for the substrate of consciousness. They add here that integrated neuronal activity cannot account for how we create and differentiate our various qualia. This important argument starts with a reference to Muller (1835), who tried to explain different qualia by saying that different sensory neurons have different “specific nerve energy.” But Adrian (1928) argued that all action potentials are the same–whether they are from visual, auditory, or other nerves. Adrian thought that what is important here is not which nerve fires but where the nerve projects to (e.g., visual or auditory cortex). However, this view was later questioned because, for example, visual cortex can support inputs from visual or auditory receptors. Here, it is the input, not the cortical receiving area, that determines qualia. This points away from neurons toward neural input from the environment.

So, this is why Ward and Guevara claim that different qualia cannot come from indistinguishable neurons but must come from different EM field structures that model the different information structures of the environment. However, more in tune with Muller above, Jones (2019) gives recent evidence that different qualia may come from different proteins in sensory detector neurons and (in the case of emotions) limbic neurons. He argues that colors may thus arise from various levels where these proteins are found–from retinas to the V4 cortical area (see below). Ward and Guevara do not address emotional qualia. This raises the issue of whether a purely thalamus-based account of qualia can account for all qualia. This is an interesting debate.

There is another interesting issue raised by Ward and Guevara’s account of integrating qualia information via EM. In their thalamic fields, each bit of information seems to be pooled indiscriminately with others, so it is unclear how these fields selectively connect information about particular colors and shapes in systematic ways–which is the integration problem (*cf.* §2.5 below).

An issue also arises in connection to Ward and Guevera’s attribution of sensory qualia to thalamic-nuclei EM fields. This attribution contrasts with the view of Neitz and Neitz (2014), who argue that retinal opponent cells actually disambiguate cone inputs and may thus be responsible for color percepts. So, retinas arguably create raw color qualia, while (as Crick and Mumford might say) thalamus arguably acts on these at higher levels that can involve attention and integration. In this way, various levels of sensory activity could be unified by coherent field activity across these circuits. Qualia–from raw to meaningful levels–would be a multi-level (nested) affair rather than being tied mostly to thalamus. This might explain why thalamic and cortical distortions do not appear in visual images, while retinal detachments and retinal blind spots do (Jones, 2019).

Ward and Guevara’s thesis that our different qualia are different “information spaces” (in Chalmers, 1996) within thalamic EM fields seems to have a similar status to IIT’s qualia theory above. A useful theory of how qualia arise needs to be testable, yet neither theory makes precise, testable predictions about how different qualia arise, which raises the coding/correlation problem above. Nor does either theory deal with the hard problem above. Like other EM-field theories of qualia, Ward and Guevara’s faces the integration problem concerning, for example, how myriad qualia are integrated to form pictorial images. In all fairness to their provocative theory, it seems to be in much the same boat as most other EM-field theories in these various regards.

### 2.3. Qualia as fundamental EM activity

While Pockett attributes qualia to macro-level EM fields, other authors try to ground qualia in fundamental-level EM-field events. These authors include Keppler, Shani, and Bond.

#### 2.3.1. Keppler and Shani

Keppler (2021) tries to develop a fundamental theory of consciousness that can fit qualia seamlessly with physical and psychological science and predict the phenomenal state of any system, given its physical state. He criticizes common claims (including those in many EM-field theories of mind) that conscious states emerge from physical complexity. For this creates troubles in explaining how consciousness can (seemingly) magically pop into existence from previously nonconscious states (Strawson, 2006), and in detailing which states this emergence occurs in.

Keppler tries to avoid this problem with a fundamental (versus emergent) theory of consciousness based on quantum theory. He notes that EM is the fundamental force shaping biological systems (*cf.* Hales and Ericson, 2022). Whereas EM dynamics are usually framed in physics as a matter of classical field physics, Keppler frames his theory of consciousness based on the quantum theory of EM fields (quantum electrodynamics or QED), which is a more fundamental physical theory than the classical approach. QED explains EM by treating the classical vacuum state as a vibrant ocean of energy, rich with structure, energy, and potentialities. Even more basic than QED is stochastic electrodynamics, which views the vacuum as an omnipresent EM background field with the lowest possible energy–the zero-point field (ZPF)–which mediates all EM events. Here, the potential energy in charged particles comes from them being embedded in the ZPF.

Keppler and Shani (2020) see the ZPF as a foundational component of the cosmos with two aspects. Its extrinsic appearance is physical, and its intrinsic manifestation is conscious. The entire palette of qualia is supposedly rooted in the ZPF’s vibrational spectrum in a potential (versus actual) way. This ZPF is thus the carrier of primordial energy and consciousness. Intriguingly, it resembles the formless sea of consciousness in mystical Hindu thought (Shani and Keppler, 2018).

They further speculate that the brain generates actual concrete (versus potential) conscious states and qualia by coupling with specific ZPF modes in resonating, oscillating ways. Here, these modes operate like a keyboard for composing various conscious states. Interestingly, this view thus “shares commonalities with the General Resonance Theory (GRT) of Hunt and Schooler (2019), according to which resonance-induced phase transitions underlie the formation of macro-conscious entities” (Keppler, 2021). In this process, oscillating cell assemblies are orchestrated by synaptic input. Here changing neurotransmitter concentrations alter the resonance properties of the assemblies “by altering their coupling strengths to synaptic action fields.”

Keppler concludes that familiar mechanisms for consciousness–such as the synchronous dynamic core of Edelman and Tononi (2000), the global neural workspace of Dehaene et al. (2006), or various EM-field views noted above–produce consciousness in mysterious emergent ways. By contrast, in his theory, a deeper, fundamental mechanism is at work in which neuronal assemblies couple in resonant ways to an omnipresent field of consciousness. This consciousness is fundamental, not emergent. This coupling process thus delineates conscious from nonconscious activities in this fundamental way. This mechanism is “the truly global workspace in which conscious processes unfold” (Keppler, 2021). The ZPF is thus a creative force behind the scenes with no equivalent in classical physics (Keppler, 2021). it is the key to this fundamental theory of consciousness.

Keppler’s theory may yield predictions about neural correlates of qualia. Subjective reports of our different qualia experiences can be linked to different neurotransmitter levels and thus to phase-locked ZPF states. Qualia spaces would thus be systematically mapped onto ZPF information spaces (Shani and Keppler, 2018; Keppler, 2021).

Keppler (2016) argues that local consciousness in the ZPF increases with the degree of phase-locking in the local ZPF–and the integration of its information. The latter parallels IIT in some ways. The two theories parallel each other in other ways too. In the end, Keppler and Shani’s approach to qualia, like IIT’s, is theoretically impressive–yet also speculative and not yet backed up with actual evidence of specific correlates for qualia. Their interesting claim about resonance’s role in creating qualia is quite credible, yet their claim that this role involves tapping the ZPF remains highly conjectural. For example, they do not detail the specific mechanisms for how this “tapping” would systematically unite shapes and colors point by point to make images.

Also, Keppler and Shani are unclear about how qualia and EM are metaphysically related. In their dual-aspect view, the ZPF’s extrinsic manifestation is an abstract mathematical structure, while its intrinsic manifestation is conscious and qualitative. These extrinsic-intrinsic and dual-aspect relations are arguably among the murkiest ones in philosophy of mind today. For these various reasons, Keppler and Shani arguably face the coding/correlation problem and the hard problem. They also seem to face the integration problem concerning how myriad qualia are integrated to form images. In other words, they face many of the problems confronting field theories in general.

#### 2.3.2. Bond

Another author who attributes qualia to fundamental EM activity is Bond (2023). This clear, succinct paper explains that quantum coherence involves the entanglement of quanta within energy fields, including the EM fields generated by neurons. Neural matter typically lacks this coherence because the haphazard orientation of quantum spins in the matter creates destructive interference and decoherence. Bond proposes the novel idea that firing neurons generate EM fields that can flow through nearby molecular structures and entangle with their atoms. This coherence produces our perceptions. The different subjective feelings of these perceptions come from different hybrids or mixtures of the fields’ wavelengths as they vibrate or resonate.

On a larger scale, this coherence ties into the well-known phase-locking of corticothalamic feedback loops. Together, they produce the holism or unity of consciousness. This combination of coherent, phase-locked feedback loops and coherent, entangled wave-particles in EM fields is called by Bond a “coherence field.” It is investigated by his Coherence Field Theory (CFT).

This CFT supplements McFadden’s well-known CEMI theory. The latter stresses that phase locking in feedback loops produce a strong EM field. This pools and integrates information in neurons, part of which is conscious. CFT adds nanoscale quantum coherence to the macroscale phase locking to explain unified consciousness. It is unclear how testable CFT is at this time.

An issue that Bond may face (if we interpret him correctly) is whether unified consciousness might be better explained in CFT simply by EM in macrolevel phase-locked feedback loops–rather than in combination with nano-level entangled wave-particles. One question here concerns how entangled wave-particles–which are no longer separate individuals describable independently–can account for all the varieties and differences in sensory experience.

Be all that as it may, one of Bond’s many exciting claims is that the complex, diverse qualia we feel could arise from enhancing the vibrations in nanoscale matter by means of the *vibrations in specially adapted macroscale neural structures*. Presumably, these structures could span all the way from specialized neuronal proteins up to cortical columns. This may have important implications for two views we sketch below–Hunt and Schooler’s (2019) attribution of qualia to resonating EM fields and Jones (2019) attribution of qualia to electrically active proteins in sensory and limbic neurons that detect sensory stimuli and hormones. Bond’s claim may point to a way of synthesizing these views (see below). Whether or not his binding by entanglement idea is right, his various views are important and will hopefully be further developed. One way his CFT stands out from other theories is its headway (as just described above) into the integration problem concerning how qualia become integrated into overall perceptions.

### 2.4. Qualia in Hunt and Schooler’s general resonance theory

Another approach to the Qualia Problem is Hunt and Schooler’s General Resonance Theory (GRT), which is grounded in a panpsychist framework (Hunt, 2011, 2014; Schooler et al., 2011; Goff, 2017; Hunt and Schooler, 2019). Hunt is a co-author of the present paper.

GRT assumes that all matter is associated with at least some capacity for phenomenal consciousness (this is called the “panpsychism axiom”), but that consciousness is extremely rudimentary in the vast majority of cases due to a lack of physical complexity mirrored by the lack of mental complexity. The EM fields associated with all baryonic matter (i.e., charged particles) are thought to be the primary seat of consciousness simply because EM fields are the primary force at the scale of life (strong and weak nuclear fields are operative at scales far smaller and gravity is operative mostly at scales far larger). Accordingly, GRT is applicable to all physical structures and as a theory is not limited only to neurobiological or even biological structures (Hunt and Schooler, 2019).

GRT suggests that resonance (similar but not synonymous with synchronization and coherence) of various types is the key mechanism by which the basic constituents of consciousness, when in sufficient proximity, combine into more complex types of consciousness. This is the case because shared resonance allows for phase transitions in the speed and bandwidth of information exchange to occur at various organizational levels, allowing previously disordered systems to self-organize and thus become coherent by freely sharing information and energy. The speed and bandwidth of information flows achieve a step change through such a phase transition, allowing for the unity of consciousness in each moment. This is GRT’s suggested solution to the binding problem as well as the Qualia Problem.

In GRT, consciousness is a product of resonance chains^4^ of various information/energy^5^ pathways, and the spatial and temporal boundaries of any particular conscious entity is established by the slowest-frequency shared resonance within that conscious entity, for each particular information/energy pathway (Hunt, 2020b). Shared resonance and resulting resonance chains are the key mechanisms for self-organization and are constantly changing in most entities (Walleczek, 2000). Thus, the spatial and temporal boundaries of conscious entities will be constantly changing at least a little (Hunt calls this constantly changing EM field structure in human and mammalian brains “the blob” in Hunt (2020b), and this structure is the physical basis for the dominant consciousness in each moment).

Most combinations of consciousness, in which less complex entities combine into more complex entities in biological structures like mammal brains, will be comprised of a nested hierarchy of conscious entities, with one dominant conscious entity in each moment, and without extinction (elimination) of the nested entities’ subsidiary consciousnesses. This notion is stated well by Whitehead et al. (1929): “The many become one and are increased by one.” This lack of extinction of subsidiary entities distinguishes the present approach from IIT and other theories that assume the extinction of nested conscious entities, leaving only one macro-conscious entity left (this is, e.g., IIT’s “exclusion principle”).

Qualia, in GRT, are synonymous with consciousness, which is simply subjective experience. Nevertheless, qualia may act as a conceptual tool for distinguishing specific qualities or aspects of consciousness. As such, some degree of qualia are associated with all EM field activity but will be more complex in more complex physical structures such as evolved biological entities with advanced sensory abilities (such as humans and other animals). Any EM field shape, which can be represented visually with the traditional EEG frequency and amplitude sine wave diagram, represents a specific quale or experience–but only at a specific level of organization. Any complex quale or moment of human consciousness, for example, is an extended nested hierarchy of resonating fields starting perhaps with extremely fast terahertz-level frequencies in microtubules and other similar subcellular proteins, and then upwards through the chain of complexity to the global EM fields measured by normal EEG at the 2–60 Hz frequency bands conventionally labeled delta through gamma.

Each layer of this extended nested hierarchy forms part of the highest-level quale or moment of consciousness, with the specific types of resonance between each level determining what information is passed from the lower level to the higher level and vice versa. The atlas of patterns comprising specific quale in any particular milieu is termed the “resonome” in GRT. The details of what comprises specific resonomes in each species have not yet been fleshed out so this term is a placeholder for now.

Chalmers (2017) asks “how do microqualities combine to yield macroqualities?” He labels this “the quality combination problem.” (We call this the integration problem.) He adds: “Here macroqualities are specific phenomenal qualities such as phenomenal redness (what it is like to see red), phenomenal greenness, and so on. It is natural to suppose that microexperience involves microqualities, which might be primitive analogs of macroqualities. How do these combine?”

GRT answers this question as follows: the oscillating/vibrating nature of all baryonic matter allows combination when achieving a shared resonance frequency between different constituents in proximity, with the speed of the specific energy/information flows that are present within each oscillation time period determining the size of the conscious entity in each moment. Biological structures have mastered the use of higher-speed information channels (nerves, electrical fields, etc.), through various types of resonance, allowing for much larger conscious entities (compared to non-biological structures) to form and to be sustained as semi-stable patterns over time. Their combination, as described above, includes the combination of their experienced qualities into a macro-conscious subject. Just as a musical note or chord is the sum of its constituents, or a paint color mixed from other colors is the sum of its constituents, the qualities (qualia) of each macro-conscious entity are the sum of its constituents in each moment.

Hunt (2020b) fleshes out a quantitative framework for GRT and allows for the calculation of the complexity of consciousness, which may be characterized as qualia in specific entities in each moment. A single quale can be calculated in GRT, in terms of its capacity for phenomenal consciousness as a scalar value, following Equation 5 from Hunt (2020b), with Ω representing the capacity for phenomenal consciousness, Δ_t_ symbolizing any specific duration, such as 1 s, 1 min, etc., and f_SSR_ the frequency of the Slowest Shared Resonance:


$$\Omega \;\left(\Delta_{t}\right)=\Omega^{*}\;f_{SSR}{}^{*}\;\Delta_{t}$$

While GRT uses the tools of information theory to calculate the capacity for phenomenal consciousness and of specific qualia in each moment, it is not a computationalist account of consciousness because it does not reduce consciousness, in an ontological manner, to information or information processing. Rather, consciousness is a fundamental feature of the fields associated with baryonic matter (and, to be precise, of any matter or fields more generally, though it appears that baryonic matter EM fields are the “main game in town” in terms of being the most suited physical structures for complex consciousness).

Hunt et al., 2022 proposes a framework for testing GRT and related theories of consciousness by measuring various “measurable correlates of consciousness” (MCC), which include Neural Correlates of Consciousness (NCC), Behavioral Correlates of Consciousness (BCC), and Creative Correlates of Consciousness (CCC).

### 2.5. Qualia as EM substances

Jones (2017, 2019), a coauthor of the current paper, has developed an EM-field theory of qualia. Like other field theories, it attributes qualia and images to neural EM-field patterns (and probably the EM-charged matter emitting the fields). Yet these are not the coded images of computational field theories that are based on information processing. Instead, in his theory images actually reside in conscious, pictorial form within the EM fields of neural maps.^6^

Admittedly, machine learning and deep learning have decoded EEG and fMRI data to infer visual images of faces and other objects (e.g., Nemrodov et al., 2018–*cf.* Lin et al., 2022; Takagi and Nishimoto, 2022).^7^ But these data appear to come from the fusiform gyrus whose processing does not (counter to some computationalists) really encode actual facial images. For the inferred faces only partly resemble the actual images.

Moreover, fusiform gyrus does not create images, it just recognizes faces as Aunt Bea, *et cetera*. Injury to this area can harm facial recognition but not the production of facial images. So, the EEG data do not encode conscious images, they encode related nonconscious processes that culminate in consciously recognizing Aunt Bea (lots of visual processing is subliminal like this).

Further, it is unclear what brain mechanism would decode the coded face (like the machine learning did to EEG patterns above). Also, how can actual images pop into existence from coded images that lack color and pictorial form? Strawson (2006) dismissed such emergence as magic. Computationalists end up with three quite different entities–abstract information, concrete EM patterns, and visual images–with unclear relations between each (Jones, 2016).

Nonetheless, field patterns might be eventually found in EEG or fMRI data from areas of the brain that create images instead of interpreting them. But Jones does not think these field patterns will have coded, nonpictorial form. In his view, images are not obscure, elusive *codes* that the brain must somehow decode. They are simply neural EM *substances* laid out in conscious pictorial form in the fields of neural maps (which are the only neural structures having pictorial arrays of color detectors).

Here, “substance” denotes the concrete, fundamental stuff comprising the universe (e.g., EM), whether it is seen as a thing or a process. Note that while Jones’s theory treats images as substances, not as computations (i.e., coded information processing), it accepts that brains refine images’ depth, constancy, etc. behind the scenes using computations (viewed simply as material interactions, not as abstract multiply realizable relations–§1.5).

This is a neuroelectrical, pure panpsychist theory of mind (NP). The “pure panpsychism” says that everything (not just EM) is comprised purely of consciousness. This partly resembles Strawson’s (2016) well-known panpsychism. The “neuroelectrical” refers to how consciousness in molecules, cells, etc. is united to form overall minds by the strong, continuous EM fields localized in ion currents along neuronal circuits (these are not global fields pervading brains, distinguishing this approach form Hunt and Schooler’s GRT). Again, images and their color qualia are EM substances laid out in neural maps. NP addresses the hard problem, qualia-integration problem, and qualia coding/correlation problem (see §1.5, §2.1.3) in the following ways.

(1) *The hard problem*: How are qualia metaphysically related to brains and computations? In NP, consciousness and its qualia are the hidden nature of observable matter and energy. We are directly aware of our inner conscious thoughts and feelings. Yet we are just indirectly aware of the observable, external world through reflected light, instruments, sense organs, *et cetera*. The world is thus hidden–its *real* nature is up for grabs. So, for all we know, consciousness may be the real, underlying nature of the external world, beyond how it appears to our senses. Here, consciousness is the world’s real, underlying substance (its concrete, fundamental stuff). It occupies space, exerts forces, and is matter-energy’s sole constituent. Physicists cannot rationally object to this view, for they describe all particles and fields solely by their observable effects–while NP refers to what particles and fields are in themselves, apart from their observable effects.

NP is arguably clearer than existing mind–body theories because it does not reduce consciousness to the observable events of physics. Also, it is simpler and clearer than computationalist and functionalist views, with their obscure relations between qualia, brains, and computations (which are abstract relations–§1.5). Only consciousness exists in NP, and it is the real, hidden nature of matter-energy. NP’s monism also avoids traditional dualism’s two different substances with their unclear causal relations. NP may also avoid various other mind–body issues (Jones, 2010, 2016).

(2) *The qualia coding/correlation problem*: How do our various qualia arise? Field theories (including Pockett’s) have not yet spelt this out. Yet there is now growing evidence that different qualia correlate with different electrically active substances in cellular membranes found in sensory and emotional circuits. These substances are the membranes’ ion-channel proteins and associated G-protein-coupled receptors (GPCRs). They detect the presence of hormones and sensory stimuli, then directly or indirectly generate electrical impulses in limbic and sensory circuits. They are thus exceedingly active electrically–both as EM-charged proteins and the EM fields they generate.

For example, the different primary colors correlate with different OPN1 GPCRs,^8^ different temperatures correlate with different TRP ion channels, and some different tastes correlate with different T1R and T2R GPCRs.^9^ These proteins reside in membrane electrical activity at various levels of the sensory system. There is also evidence that oxytocin and vasopressin receptor proteins correlate with feelings of love (e.g., Busnelli and Chini, 2018).^10^^,^^11^ Also, estrogen and testosterone receptors correlate with lust (Fisher, 1997), the endorphin receptor correlates with euphoria (e.g., Sprouse-Blum et al., 2010), and the adrenaline receptor correlates with vigilance (e.g., Bayerl and Bosch, 2019). Jones (2019) gives a much longer list of correlations between sensory qualia and proteins, with supporting arguments and citations.

**Figure 2:**
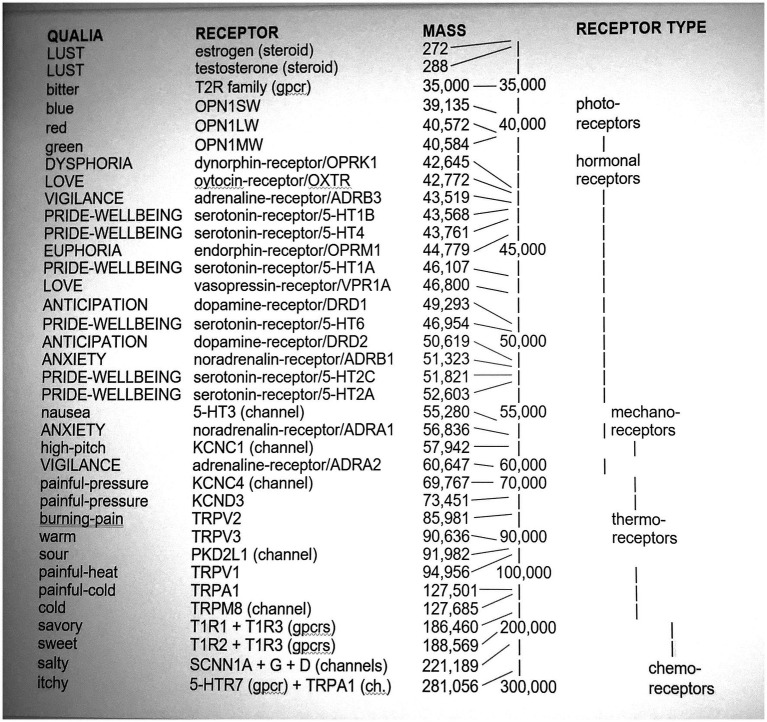
Protein Correlates of Sensory and Emotional Qualia: Molecular biology is finding growing evidence that neural proteins correlate with our qualia. Column 1 lists sensory and emotional qualia in lowercase and uppercase, respectively. Column 2 lists the correlating proteins–usually ion channels or GPCRS that detect sensory stimuli or act as hormone receptors–all in electrically active ways. Column 3 lists (at a more fundamental level) the proteins’ masses (in Dalton units). This column 3 shows that each of these electrically bound proteins has a distinctive mass–and thus distinctive rest energy (which Jones, 2019 construes as the protein’s fundamental substance). While some masses (m) are fairly close, their rest energies (mc^2^) lie exponentially far apart. Finally, note that some qualia correlate with more than one mass (which is unsurprising because these qualia likely reside like repeating rainbows in the range of electrically bound masses in nature). Yet each mass correlates (crucially) with a different quality. This figure comes largely from papers and directories (e.g., genecards.org/) cited in Jones (2019). Evidence for the emotional qualia is sometimes less conclusive than with the sensory qualia.

Jones (2019) thus identifies qualia with these proteins. In his view, Figure 2 below is the only existing list of neural correlates for qualia.^12^ He argues that neuroplasticity does not threaten this list.^13^ His identification of qualia with specific proteins is partly testable, for it predicts that the qualia-protein correlations in Figure 2 are not flukes and will continue expanding to eventually include all qualia.

Returning to color qualia and visual images, they may reside in the resonating EM fields of opsin proteins (and in a fundamental way, not in a problematic emergent way).^14^ These opsins may thus form the labeled lines for colors, while cross-line comparisons modulate which lines are most active. (For example, we see blue when long-wavelength lines are activated and when opponent cells inhibit the other two opsin lines–all in line with existing theories of perception.) In contrast, other field theories have not yet been able to specify field patterns that encode qualia.

(3) *The qualia-integration problem*: First, how do various qualia unify together into an overall whole? Second, how specifically do qualia join point by point to form pictorial images?

First, neuron-based theories have trouble explaining this *unity*, while field-based theories excel here. In NP’s field theory, active circuits create a continuous EM field between neurons that pools their separate, atomized consciousness. This creates a unified conscious mind along brain circuits (with the mind itself residing in the field and perhaps in the charged matter creating the field). This unity is strongest around the diffuse ion currents that run along (and even between) neuronal circuits. It is very strong among well-aligned cortical cells that fire together coherently. Yet this field degrades exponentially with distance, which can explain why consciousness is not united between brains and why minds are private. Even within each brain, the field is at times too weak to fully unify consciousness, leaving much brain activity merely subliminal.

Evidence that unified cognition comes from EM takes three forms. (A), no other mechanisms seem to explain the mind’s unity (§1.3). (B), Koch et al. (2016) argue that locally activated EEGs actually track conscious perceptions across brains better than other events, such as neuronal firing synchrony or P300 events. This EEG evidence links perceptions (i.e., unified sensory experiences) to local neuroelectrical fields. (C), EM fields–rather than just particles or synapses–propagate signals across slices in hippocampal tissue (Chiang et al., 2019; *cf.* Libet, 1993). This indicates that it is most likely the fields that unify this activity.

Second, neuron-based theories, as discussed above, also have trouble explaining how we see overall *pictorial images*. For we lack top-level detectors to encode all the possible scenes comprising our pictorial images. Some field-based theories have trouble here too. Their difficulty is in showing how EM fields (which lack the specificity of neuronal connections–except, as discussed above with respect to Hunt and Schooler’s GRT–through selective resonance) can systematically attribute colors point by point all across images. As Pockett’s account illustrates, it is difficult to distinguish color and spatial information in fields. Jones suggests that no EM-field patterns have yet been found that actually encode the creation of images (versus associated events such as facial recognition).^15^ Nor is it clear how to get from these codes to the actual conscious images.

In NP, images are not obscure, complicated coded activities. Instead, they are simply arrays of electrically active detectors laid out in pictorial form across neural maps. All the intense electrical activity of these maps is fully conscious–from retinas to the thalamocortical areas that they tie into.^16^ Their images are not separate, for their systematic electrical connections bind them point by point into a single unified conscious whole.^17^

Retinal opsins and cones feed into numerous V1 color processors (blobs), which in turn activate V4 color processors and flood their opsins with currents. This helps V1 to create detailed colors, V4 to create color constancy, and retinas to create images’ pictorial form and overall elliptical shape at their peripheries. Damage to V4 blocks colors from accessing higher cortical levels that support the overall unified mind with its controlling subject. So, color blindness results.

NP might ultimately attribute pictorial images to the standing waves in retinas and other neural maps that connect to them (*cf.* Lehar, 2003). These waves are like the patterns of ripples across fluid surfaces in vibrating containers. They are standing in that they are created by stable map structures. The spatial layouts of retinal standing waves would come from arrays of cone activity. This would provide the pictorial layouts of images. Different colors would be different local field perturbations generated by the EM dynamics of each opsin folding and unfolding–and the intense, oscillating ion currents this unleashed.

In summary, computational field theories have not yet shown how to encode the colors and pictorial form of images, and they are unclear about how to get from any such codes to conscious images. NP treats images not as neural codes but as neural substances–the pictorial standing waves of neural maps, beyond how they appear to EEGs. This theory is partly testable.

So, NP ends up differing from many other EM-field theories of qualia. Everything is conscious in NP, not just EM fields. Also, minds are unified by local EM fields right around neural circuits, not by global fields pervading brains. Nor are qualia encoded in field patterns, instead they are laid out in pictorial form across EM fields. Finally, qualia are not emergent from, nor intrinsic to EM, but are the real nature of EM beyond how it appears EEGs. NP is perhaps closest to the GRT of Hunt and Schooler. above, especially when it comes to GRT’s account of the multi-scale EM fields associated with brains as the primary seat of consciousness, with the brain as a relatively stable underlying neuroanatomical backbone supporting conscious EM fields. GRT supporters might not agree with NP’s attempt to extend GRT’s approach to qualia. In the end, NP arguably mixes neuronal and field approaches to qualia, for qualia and images reside in the neural EM field, and perhaps also in the charged neuronal matter that generates this field.

NP’s drawbacks are its conflicts with other field theories. Jones thinks these differences are justified, but other field theorists often disagree. Especially contentious are NP’s pure panpsychism, its local fields, its anti-computationalism, and its claim that all qualia correlate with neural proteins.

## 3. Conclusion

Consciousness is characterized mainly by its privately experienced qualities (qualia). Standard, computation-based and synapse-based neuroscience have serious difficulties explaining them. Key commonalities between consciousness and EM fields led us to review EM-field theories of qualia to see if they can improve upon standard neuroscience’s approaches to three crucial issues. (1) What neural events encode or correlate with the various qualia? (2) How do neural events integrate qualia to form (for example) pictorial images? (3) Are neural events metaphysically related to qualia by identity, causality, third entities–or some other relation? We call these the qualia coding/correlation problem, the qualia-integration problem, and the hard problem, respectively.

(1) Field theories usually look for different field patterns that encode or correlate with different qualia. But they have not yet established that such patterns exist. Nor do they agree on whether to construe these patterns as *codes* (information processing) or as *substances* (physical stuff). One option is to continue looking for patterns that encode qualia. For example, Nemrodov et al. (2018) argue that EEG studies of face processing in brains show the “rich informational content of spatiotemporal EEG patterns.” Another, less recognized option (suggested in Jones’s work) is to look for correlations between qualia and certain EM substances, such as the vibrating fields (and charges) of certain proteins (e.g., colors seem to correlate with the EM activity of wavelength detectors–opsins). It is presently unclear whether these options will improve upon standard neuroscience.

(2) Field theories have arguably made real progress in explaining how fields integrate colors to form unified pictorial images. This unity comes not from field codes, but from the continuous extension of EM fields across space. This extension allows neurally-associated EM fields at various spatiotemporal scales to pool qualia together to create a single, unified consciousness. But field theories must make more progress in explaining how fields integrate qualia, such as colors, point by point across space to make (in this example) pictorial images. One option is to continue looking for how field patterns encode spatial arrays. Another is to look for pictorial standing EM waves (like those in the fluid surfaces of vibrating containers) in neural maps rooted in retinas. Here, images are the substances of EM activity in pictorially arranged visual detectors.

(3) Field theories are in the same situation as standard theories concerning the hard problem of whether neural events are metaphysically related to qualia by identity, causality, or some other relation. But field theories do employ fairly recent metaphysics (such as Whitehead’s, Strawson’s, Shani’s, and McFadden’s) that can protect them from traditional mind–body problems. GRT and NP, for example, both suggest that an intrinsic property, or the true nature, of EM fields is qualia/conscious experience.

So, field theories have improved in key ways upon standard neuroscience in explaining qualia. But this progress is sometimes tentative–it awaits further evidence and development.^18^

## Author contributions

MJ wrote the draft. TH provided numerous rounds of revisions and feedback. All authors contributed to the article and approved the submitted version.

## Conflict of interest

The authors declare that the research was conducted in the absence of any commercial or financial relationships that could be construed as a potential conflict of interest.

## Publisher’s note

All claims expressed in this article are solely those of the authors and do not necessarily represent those of their affiliated organizations, or those of the publisher, the editors and the reviewers. Any product that may be evaluated in this article, or claim that may be made by its manufacturer, is not guaranteed or endorsed by the publisher.

## Footnotes

^1^The qualia problem is related to but different than the problem of memory (how are memories encoded, stored and retrieved), because memories could in theory be encoded without qualia. There is no apparent necessity that recording information about the external world would entail affective components. Indeed, we do not assume that a video camera recording successive images of the world includes any affective component in the camera or the images it takes. And yet our memories of the world–akin to snapshots stored in our brains–seem to always include affective components, at least initially. The same is true of our reactions in real-time to the world. It may turn out that the qualia problem and the “memory problem” are the same problem, if indeed memories cannot be encoded without affect.

^2^Such experience is usually called “phenomenal” or “qualitative” consciousness to contrast it with “access” consciousness, which is the availability of information for acting, speaking, reasoning, *et cetera*. But the latter arguably has no essential connection with qualitative experience and is thus not consciousness at all.

^3^It may seem that accounts of binding (unity) are irrelevant to this paper’s basic topic of qualia. But the two are inseparable. For example, colors are always experienced in visual space where they are bound together as unified images. Also, as the account of qualia theories will illustrate below, these theories get into trouble when they try to explain how different qualia arise without looking at how they bind together in images (e.g., see Pockett in §2.1.3).

^4^Bandyopadhyay has developed a sophisticated approach to resonance chains in a broad theory of consciousness he calls the Fractal Integrated Information (FIT) theory of consciousness (Bandyopadhyay, 2019). Resonance in GRT is similar to its role in FIT, but GRT adopts a metaphysically foundational role for resonance through its general congruence with Whitehead’s process philosophy and the “actual entities” that are the “final real things” that comprise the world (Whitehead et al., 1929; Hunt and Schooler, 2019).

^5^Information is generally defined as a subjective aspect of the physical world, whereas energy is an objective aspect; but in the context of GRT these terms are often used interchangeably because information is defined as “aspects of energy that we can measure.” Accordingly, information is a concept that supervenes on the presence of matter. All physical dynamics consist of nothing more than energy flows, but those energy flows that we can measure may be labeled “information” and may be usefully quantified under established information theoretic concepts. Hunt (2020b) offers an information theoretic framework for quantifying the presence and complexity of consciousness in any physical structures.

^6^This section represents the views of Jones only.

^7^Nemrodov et al. (2018) use EEG data while Lin et al. (2022) and Takagi and Nishimoto (2022) use fMRI data. But they all have very similar problems.

^8^Cone cells contain OPN1 proteins that are GPCRs of the opsin class. Light-absorbing molecules attached to these different opsins have different spatial conformations that detect different light wavelengths. This starts cascades of reactions, mediated by transducin molecules, which (via inward currents) hyperpolarize the cells. Depolarization quickly follows. The opsins are flooded by these strong ion currents that they trigger. It is possible here that colors may be determined not only by opsins but also by adjacent active proteins that they strongly interact with (see Jones, 2019). But the details are unclear, so this paper sticks to what seems relatively clear at present–the color-opsin link.

^9^ https://www.genecards.org/

^10^The recent discovery that genetically altered prairie voles who lack oxytocin can still pair bond normally (Berendzen et al., 2023) shows that pair bonding does not require oxytocin. But this does not threaten Jones’ claim that love requires oxytocin. Indeed, the authors argue that these altered voles may well have compensated for their lack of oxytocin by activating vasopression pathways to preserve the feeling of love and pair-bonding behavior.

^11^It may seem simplistic to attribute the vast complexities of love to the simple oxytocin molecule. But keep in mind that feelings of love are tinged with other emotions with different hormonal sources, such as joy, lust, jealousy, and anguish. Also, equating love and oxytocin is only meant to account for the innate emotional feeling (sheen) of love experiences, not the crucial conceptual components of love experiences, which are highly complex and learned.

^12^In this list, several proteins might conceivably be the same blue color. Yet one protein cannot be both blue and red. For the qualia correlations pertain only to primary qualia, which means a protein can be red or blue, but not a purple blend. Since a protein can thus only be a single quale, there’s still genuine selectivity to qualia/protein correlations.

^13^It might be argued that neuroplasticity threatens this account of qualia. For example, if visual cortex is recruited for somatosensory processing by blind subjects, and these cortical detectors are stimulated, then subjects report somatic qualia (Ptito et al., 2008). This threatens Jones’ claim that visual-detector proteins correlate with visual qualia. In reply, his view is not threatened if neurogenesis and plasticity yield not only new detector synapses, but also new detector GPCRs and channels. Many somatosensory GPCRs and channels already exist in occipital and parietal lobes (Su et al., 2004), so neurogenesis of more of them would hardly be surprising.

^14^What exactly is it about these opsins that gives them their different colors? (a) Arguably, it is their different molecular structures (electrical bonding structures). But this makes colors emergent. Blue would magically pop into existence from what lacked blue as trichromatic vision evolved at molecular levels (recall §2.3.1) So, these structures do not appear in Figure 2 below, which lists known correlates of qualia. (b) Opsins could instead get their colors from the different resonances of these proteins and their EM fields. These resonances might be construed as fundamental instead of emergent due to the fundamental energy levels they bear. But these resonances are presently unknown and thus do not appear in Figure 2 below. (c) What gives opsins their colors could also be these proteins’ different masses (m)–and thus their rest energies (mc^2^). In this case, the whole range of our qualia would reside like a rainbow in the range of these protein rest energies (in nature at large, this rainbow might repeat across many orders of magnitudes of rest energies in electrically bound masses, see Jones, 2019). These options all align with NP’s view that qualia are fundamental substances (*cf.* Keppler and Shani above).

^15^Hunt does not agree that EM field patterns do not encode images, due to a number of published studies illustrating how machine learning AI has successfully decoded various images and other qualia from EEG and fMRI signals (Nemrodov et al., 2018; Lin et al., 2022).

^16^There is evidence that colors do not exist just at cortical levels. Mancuso et al. (2009) injected genes for long-wavelength cones into monkey retinas that had only short and medium-wavelength cones (and associated opsins). The retinas rapidly grew long-wavelength cones, enabling the monkeys to discern color trichromatically. The rapid growth indicates that no cortical rewiring was involved, the authors argue. Also, Neitz and Neitz (2014) argue that retinal opponent cells actually disambiguate cone inputs, thus giving retinas the ability to create accurate color percepts.

^17^In contrast to these sensory images, the pictorial form of mental images likely comes from the undistorted visual arrays of grid and place cells in the entorhinal cortex and hippocampus. They help in imagining and navigating scenes.

^18^EM approaches may also help explain higher cognition. For example, Hunt and Jones’ “Where is consciousness” (under review) argues that higher cognition and consciousness rely on coherent EM field activities of various frequencies that perform various tasks. Additionally, Jones’s “A simple, testable, mind–body theory” (under review) gives evidence that neuroelectrical activity generates qualia, binds them into the unified experiences, helps focus attention and guide cognitive activity and metacognition, and helps form the mind’s subject (controlling center).
